# MiR-200b/200c/429 subfamily negatively regulates Rho/ROCK signaling pathway to suppress hepatocellular carcinoma metastasis

**DOI:** 10.18632/oncotarget.3700

**Published:** 2015-03-30

**Authors:** Chun-Ming Wong, Lai Wei, Sandy Leung-Kuen Au, Dorothy Ngo-Yin Fan, Yuan Zhou, Felice Ho-Ching Tsang, Cheuk-Ting Law, Joyce Man-Fong Lee, Xianghuo He, Jue Shi, Carmen Chak-Lui Wong, Irene Oi-Lin Ng

**Affiliations:** ^1^ Department of Pathology and State Key Laboratory for Liver Research, Li Ka Shing Faculty of Medicine, The University of Hong Kong, Hong Kong, China; ^2^ Department of Physics and Department of Biology, Centre for Quantitative Systems Biology, Hong Kong Baptist University, Hong Kong, China; ^3^ State Key Laboratory of Oncogenes and Related Genes, Shanghai Cancer Institute, Shanghai Jiao Tong University School of Medicine, Shanghai, China

**Keywords:** hepatocellular carcinoma, miR-200 family, cytoskeletal reorganization, Rho/ROCK signaling pathway, cancer metastasis

## Abstract

MiR-200 family is an important regulator of epithelial-mesenchymal transition and has been implicated in human carcinogenesis. However, their expression and functions in human cancers remain controversial. In the work presented here, we showed that miR-200 family members were frequently down-regulated in hepatocellular carcinoma (HCC). Although all five members of miR-200 family inhibited ZEB1/2 expression in HCC cell lines, we showed that overexpression only of the miR-200b/200c/429 subfamily, but not the miR-200a/141 subfamily, resulted in impeded HCC cell migration. Further investigations led to the identification of RhoA and ROCK2 as specific down-stream targets of the miR-200b/200c/429 subfamily. We demonstrated that the miR-200b/200c/429 subfamily inhibited HCC cell migration through modulating Rho/ROCK mediated cell cytoskeletal reorganization and cell-substratum adhesion. Re-expression of miR-200b significantly suppressed lung metastasis of HCC cells in an orthotopic liver implantation model *in vivo*. In conclusion, our findings identified the miR-200b/200c/429 subfamily as metastasis suppressor microRNAs in human HCC and highlighted the functional discrepancy among miR-200 family members.

## INTRODUCTION

Hepatocellular carcinoma (HCC) is a prevalent malignancy and ranks the third most fatal cancer worldwide [1]. However, the molecular mechanisms underlying the development and progression of HCC remain poorly understood. MicroRNA (miRNA), a class of endogenous small non-coding RNA, has increasingly been recognized as an important post-transcriptional gene regulator. In the past decade, thousands of miRNAs have been discovered and annotated in the human and vertebrate genomes. These miRNAs contribute to the regulation and fine-tuning of almost all major cellular processes, such as cellular differentiation, proliferation, migration and apoptosis [2]. Aberrant miRNA expression has been linked to various disease conditions, including cancers. In human HCC, miRNA deregulation is an early event in liver carcinogenesis and accumulation of miRNA deregulation contributes to disease progression and metastasis [3, 4].

MiR-200 family has recently been identified as a major regulator of epithelial-mesenchymal transition (EMT). MiR-200 family consists of five evolutionarily conserved members located in two separate clusters in the human genome, with miR-200b, miR-200a, and miR-429 mapped to chromosome 1p36 (thereafter, referred as 1p36 cluster), and miR-200c and miR-141 mapped to chromosome 12p13 (henceforth, indicated as 12p13 cluster). MiR-200 family can also be sub-divided into two subfamilies according to their seed sequence homology, with miR-200a and miR-141 as a group (hereafter, denoted as miR-200a subfamily) and miR-200b, miR-200c, and miR-429 as another group (henceforward, named as miR-200b subfamily) (Supplementary Figure 1a) [5, 6]. These miR-200 family members negatively regulate the expression of transcription repressors ZEB1 and ZEB2. Loss of miR-200 family results in up-regulation of ZEB1 and ZEB2 and consequently leads to transcriptional repression of E-cadherin to facilitate EMT [5-7]. Interestingly, ZEB1 and ZEB2 are also involved in the transcriptional repression of the miR-200 family, thus the miR-200 family and ZEB1/2 form a double-negative feedback loop to regulate each other. The homeostatic expression between miR-200 family and ZEB1/2 is critical for maintaining the stability of epithelial or mesenchymal phenotype of cells. Aberrant expression of either miR-200 family or ZEB1/2 will therefore feed-forward to disrupt the equilibrium and result in epithelial-mesenchymal transition (EMT) or mesenchymal-epithelial transition (MET) [8, 9]. Since EMT is a crucial step in cancer metastasis, the miR-200 family members have been considered as metastasis-suppressive miRNAs. Nevertheless, the expression of the miR-200 family in human cancer remains contentious. Both down- and up-regulations of miR-200 family have previously been reported [10-14]. These inconsistent findings are speculated to be related to the heterogeneous nature of human cancers [15]. Also, the miR-200 family may play different roles in different cancers depending on their specific cellular contexts [15]. However, knowledge regarding the expression and functional implications of miR-200 family in human HCC is awaited for detailed elucidation.

## RESULTS

### Frequent down-regulation of miR-200 family in human HCCs

**Figure 1:**
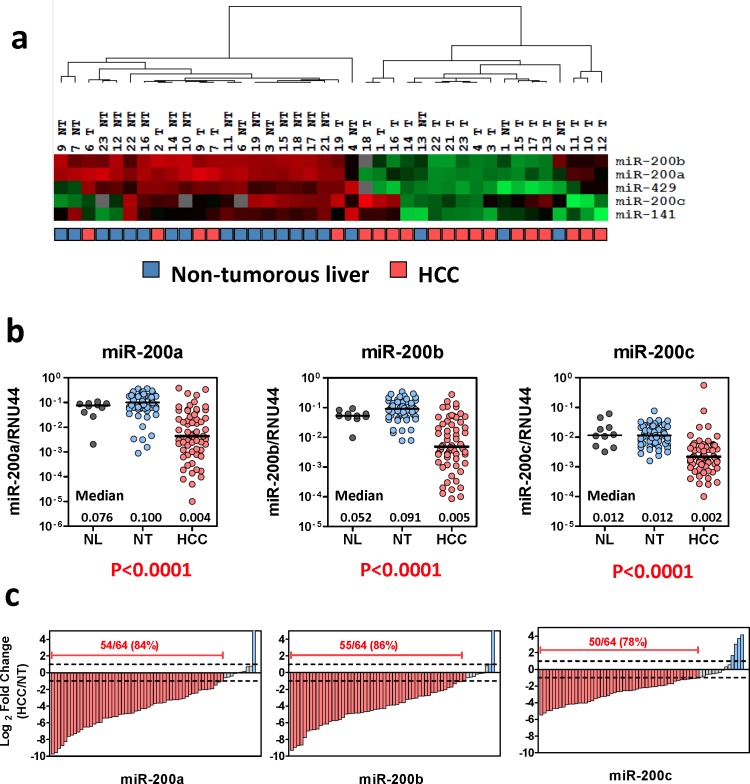
Frequent down-regulation of miR-200 family in human HCCs (**a**) Expression of the miR-200 family members in 20 pairs of primary HCCs and their corresponding non-tumorous livers. Data were retrieved from TaqMan-Low density array miRNA profiling study [4]. Relative miRNA expression levels are presented in a heat-map diagram generated by GeneCluster and Treeview software. Unsupervised clustering analysis revealed a profound differential expression of the miR-200 family members in primary HCCs. (**b**) qRT-PCR analysis performed on an expand HCC cohort (n = 64) confirmed the significant down-regulation of the miR-200 family in human HCCs (*P* < 0.001, Wilcoxon test). NL: normal livers; NT: non-tumorous livers; HCC: primary hepatocellular carcinoma. (**c**) Down-regulation of the miR-200 family (> 2 fold) was detected in 78%-86% of primary HCCs.

To interrogate the expression of miR-200 family members in human HCCs, we retrieved the data from our previous miRNA expression profiling of 20 pairs of primary HCCs and their corresponding non-tumorous livers [4]. As illustrated by the heat map diagram, all five members of miR-200 family were profoundly down-regulated in human HCCs, suggesting that loss of miR-200 family might play a role in liver carcinogenesis (Figure 1a). We reasoned that, at the expression level, the miR-200 family can be categorized into two groups based on the chromosomal locations in which two different up-stream promoters are involved (Ch. 1p36 cluster and Ch. 12p13 cluster), whereas, at the functional level, the miR-200 family members are categorized into two other groups based on the sequence homology hence two distinct groups of targets involved (miR-200b subfamily and miR-200a subfamily) (Supplementary Figure 1a) [5, 6]. Therefore, as proof-of-concept, miR-200a, miR-200b, and miR-200c were selected to represent each subgroup for expression and functional studies (Supplementary Figure 1b). We performed quantitative RT-PCR (qRT-PCR) to validate the expression of miR-200 family members in an expanded HCC cohort (n = 64) and found that, consistent to our miRNA profiling data, miR-200a, miR-200b, and miR-200c were all significantly down-regulated in primary HCC samples (Figure 1b, P < 0.001). Indeed, down-regulation (> 2-fold) of all the miR-200 family members was detected in approximately 80% of primary HCC cases, suggesting that loss of miR-200 expression was a common event in human HCCs (Figure 1c).

### MiR-200b subfamily suppressed HCC cell growth and metastasis

By comparing the expression of miR-200 in a pair of HCC cell lines derived from the primary tumor and its portal vein metastasis of the same patient, we noted that the miR-200 family members were significantly down-regulated in the metastatic cell line (H2M) when compared to its non-metastatic counterpart (H2P), suggesting that loss of miR-200 family might play a role in HCC metastasis (Supplementary Figure 2). In line with this observation, we found that the expression levels of the miR-200 family in a panel of HCC cell lines showed an opposite trend with the E-cadherin expression, and transient overexpression of miR-200a, miR-200b, and miR-200c precursors were all able to suppress the ZEB1/2 expression. These findings recapitulated the previous reports and supported the negative regulatory functions of miR-200 family in ZEB1/2 mediated EMT (Supplementary Figure 3).

**Figure 2:**
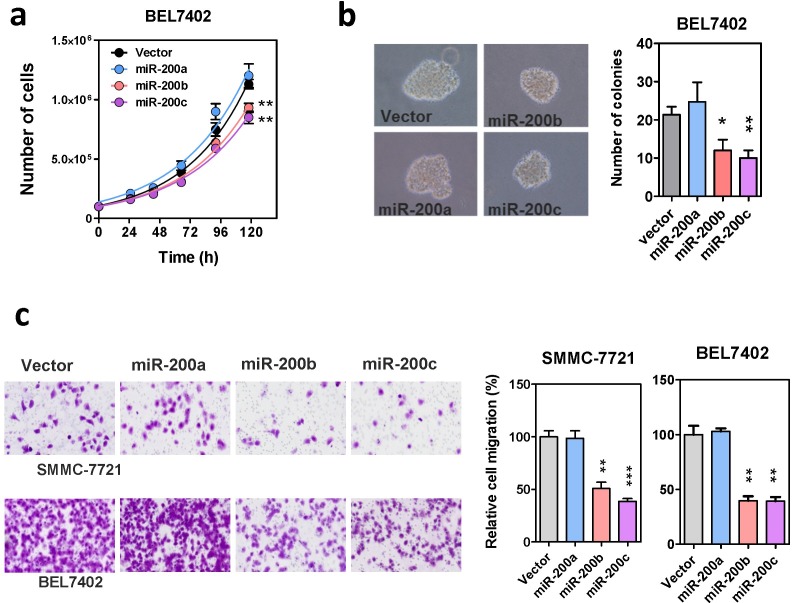
Effects of the miR-200 family on HCC cell growth and migration (**a**) Proliferation rate of miR-200 stably overexpressing cells. Cell number was counted by automatic cell counter in five consecutive days. Overexpression of miR-200b and miR-200c modestly suppressed HCC cell proliferation. (**b**) Anchorage independent growth of miR-200 stably overexpressing cells was assessed by colony formation assay in soft-agar. Both the size and number of colonies formed were significantly reduced in miR-200b- and miR-200c-overexpressing cells. (**c**) The effect of stable miR-200 overexpression on HCC cell migration in Transwell cell migration assay. Migrated cells were stained with crystal violet and counted under microscope. *P*-values were determined by *t*-test and comparing with the vector control, *P < 0.05, **P < 0.01, ***P < 0.001.

To further investigate the functions of miR-200 family in human HCC, miR-200a, miR-200b, and miR-200c were subcloned into a lenti-viral based expression vector (Supplementary Figure 4a) and stably overexpressed in HCC cell lines, BEL7402 and SMMC-7721. The successful overexpression of mature miR-200s in these stably infected cells was validated by GFP marker and qRT-PCR (Supplementary Figure 4b). In addition to the expression level, the post-transcriptional gene repressive functions of these ectopically expressed miR-200s were also confirmed by specific luciferase-tagged miRNA sensors (Supplementary Figure 4c). With these miR-200s stably overexpressing cells, we then investigated the effects of miR-200 family in HCC cell growth. Overexpression of miR-200b and miR-200c, but not miR-200a, resulted in a moderate but significant reduction on cell proliferation and also impeded anchorage independent growth on soft agar (Figure 2a and 2b). Surprisingly, we found that although miR-200a, miR-200b and miR-200c all attenuated ZEB1/2 expression in HCC cells, only stable overexpression of miR-200b and miR-200c remarkably suppressed HCC cell migration. The migration rate of HCC cells overexpressing miR-200a, was similar to the vector control (Figure 2c). This observation was reproducible by transiently overexpressing the miR-200 precursors in the same cell lines, thus ruling out the possibility of experimental artifacts that might be introduced by the lenti-viral vector or stable infection (Supplementary Figure 5). Altogether, these findings suggested that the miR-200a and miR-200b subfamilies acted differentially, with the miR-200b subfamily members specifically functioning as tumor suppressors in HCC.

### MiR-200b subfamily negatively regulated RhoA and ROCK2

**Figure 3:**
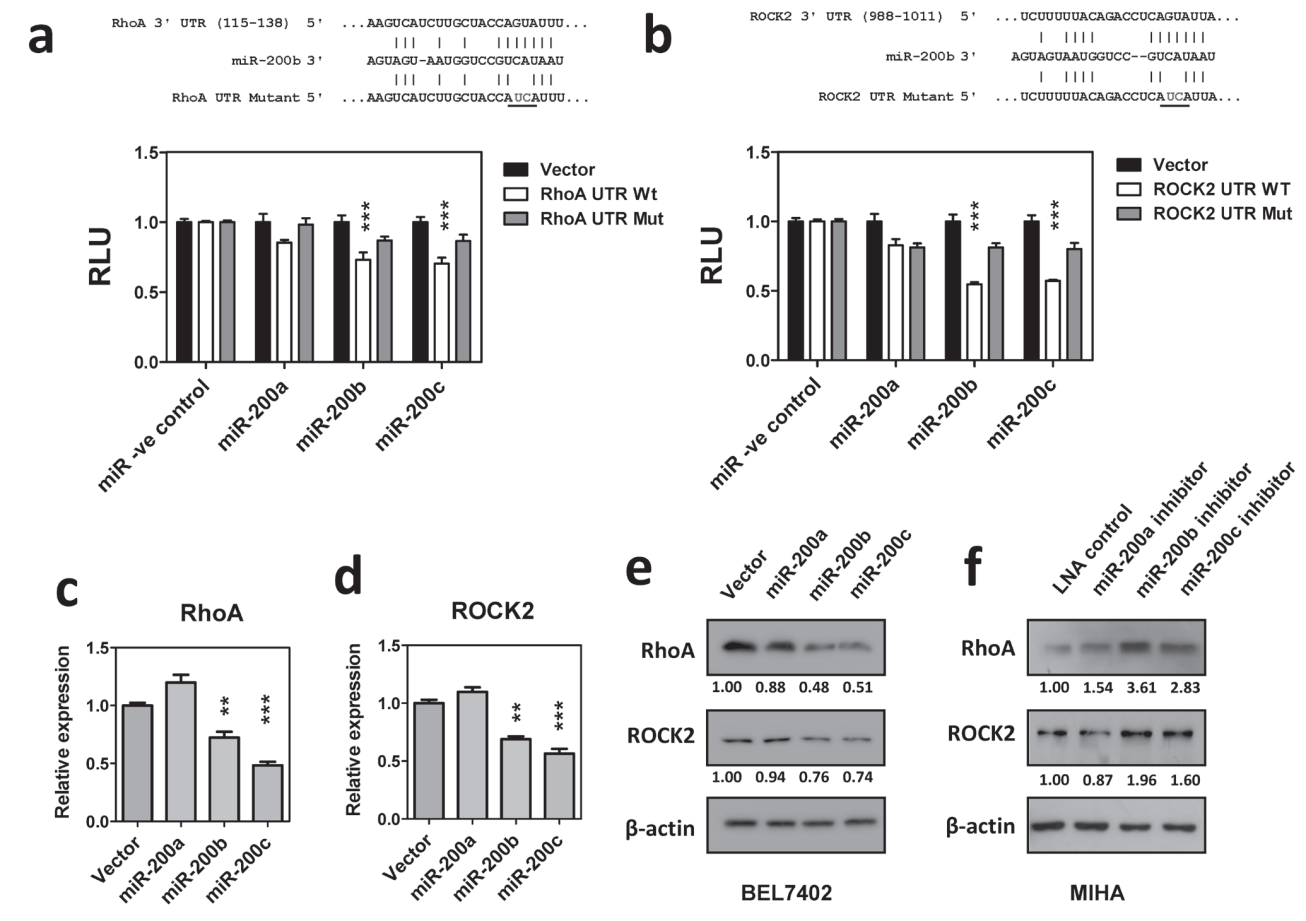
MiR-200b subfamily negatively regulated RhoA and ROCK2 expression (**a**) and (**b**) Luciferase reporter assays. Wild-type and mutated RhoA or ROCK2 3′UTR were co-transfected with miR-200 precursors into BEL7402 cells. Overexpression of miR-200b and miR-200c significantly inhibited the luciferase activity associated with the wild-type 3′UTRs of RhoA and ROCK2. RLU: relative luciferase activity normalized with the empty miRNA luciferase reporter vector control. (**c**) and (**d**) qRT-PCR analysis showed that stable overexpression of miR-200b and miR-200c significantly inhibited endogenous RhoA and ROCK2 mRNA expression in BEL7402. (**e**) The endogenous RhoA and ROCK2 protein expression levels were reduced in miR-200b and miR-200c stably overexpressing cells. (f) Transient expression of miR-200 LNA inhibitors augmented endogenous RhoA and ROCK2 protein expression in immortalized hepatocyte cell line, MIHA. *P*-values were determined by *t-*test and compared to the corresponding vector control. *P < 0.05, **P < 0.01, ***P < 0.001.

The distinct function of miR-200a and miR-200b subfamilies on HCC cell growth and migration implied that miR-200b subfamily exerted its tumor suppressive functions via a mechanism beyond the inhibition of ZEB1/2 mediated EMT. To further investigate the down-stream targets and molecular pathways specifically regulated by miR-200b subfamily, *in-silico* analysis was performed by using the EIMMO miRNA prediction server [16]. Among the 33,410 mRNA transcripts included in this analysis, 871 and 1488 mRNAs were predicted to harbor evolutionarily conserved binding site(s) of miR-200a and miR-200b subfamilies, respectively. Gene ontology (GO) analysis further revealed that the down-stream targets of miR-200a and miR-200b subfamilies were enriched in different GO terms (Supplementary Table 1). Interestingly, we noted that the down-stream targets of the miR-200b subfamily were significantly enriched in cytoskeleton genes and participated in small GTPase mediated signal transduction (P = 1.15 × 10^−5^ and 4.30 × 10^−3^, respectively). Among all, two major components of the cytoskeletal regulatory pathway, RhoA and ROCK2, were suggested as specific targets of the miR-200b subfamily by different miRNA target prediction algorithms (Supplementary Figure 6). To experimentally validate these *in-silico* prediction results, 3′UTRs of RhoA and ROCK2 were cloned into a luciferase reporter construct and co-transfected with miR-200 precursors in BEL7402. Luciferase reporter assays showed that overexpression of miR-200b or miR-200c precursors significantly suppressed the luciferase signals of RhoA- and ROCK2- 3′UTR fusion reporters. These suppressive effects were significantly reduced in the miR-200a overexpressing cells or upon mutation of the miR-200b subfamily binding sequence, thus confirming the specificity of the miR-200b subfamily in regulating RhoA and ROCK2 expression (Figure 3a and 3b). In line with this observation, the endogenous expression of RhoA and ROCK2 mRNA was significantly inhibited in miR-200b- and miR-200c-stably overexpressing BEL7402 cells when compared to the empty vector and miR-200a-stably overexpressing controls (Figure 3c and 3d). Similar findings were also observed at the protein levels as demonstrated by Western blotting (Figure 3e). Furthermore, we employed locked nucleic acid (LNA) miRNA inhibitors to specifically inhibit the expression of the miR-200 family members. Consistently, inactivation of miR-200b and miR-200c resulted in up-regulation of endogenous RhoA and ROCK2 protein expression in immortalized hepatocyte cell line, MIHA (Figure 3f). The above findings thus confirmed that miR-200b subfamily specifically targeted RhoA and ROCK2 3′UTRs and negatively regulated their expression at both mRNA and protein levels.

### MiR-200b subfamily suppressed Rho/ROCK mediated cytoskeletal reorganization and cell motility

**Figure 4:**
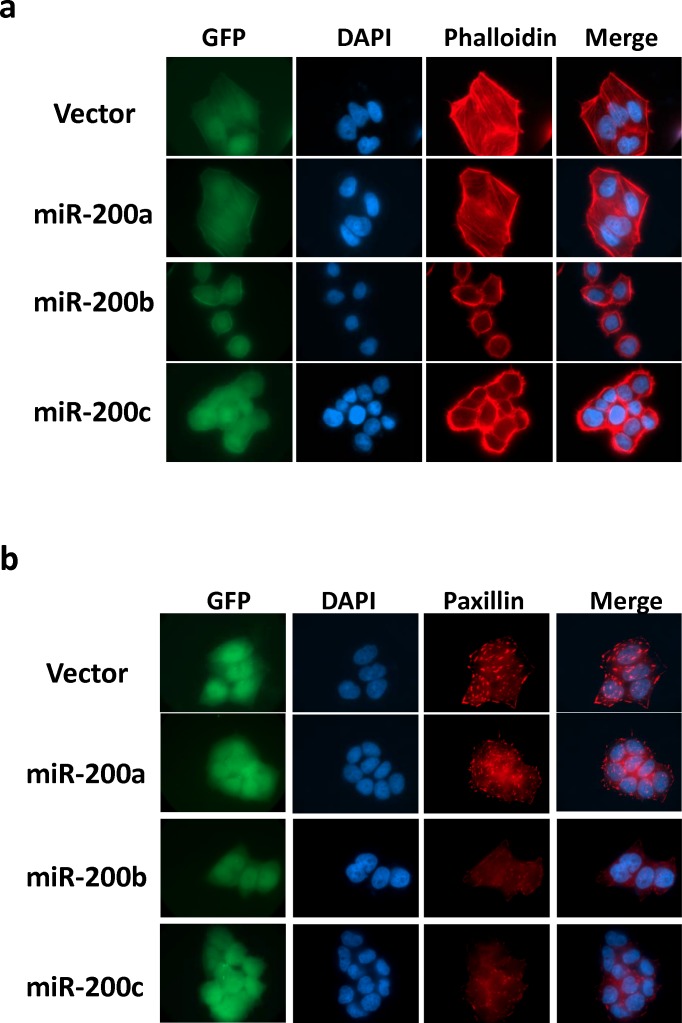
MiR-200b subfamily suppressed stress-fiber and focal adhesion formation mediated by Rho/ROCK in HCC cells (**a**) Stress-fibers (filaments across the cytosol) in miR-200 stably overexpressing BEL7402 cells were visualized by TRITC-Phalloidin staining. (**b**) Focal adhesion complexes were stained up by anti-paxillin antibody. Stable overexpression of miR-200b and miR-200c profoundly abolished the stress-fiber and focal adhesion formation and induced morphological change in BEL7402 cells. GFP maker was used to indicate the stable infection of miR-200 expression vectors. DAPI was used to counterstain the nuclei.

**Figure 5:**
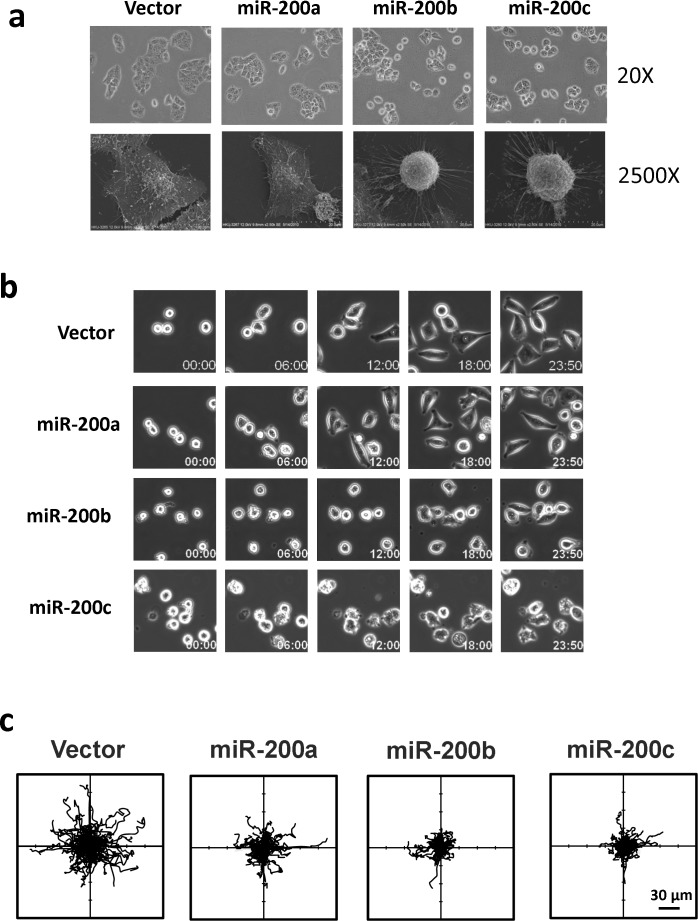
The miR-200b subfamily induced morphological change, attenuated cell-substratum adhesion and impeded cell migration in HCC cells (**a**) Cell morphology of miR-200 stably overexpressing BEL7402 cells was observed under phase-contrast microscope (20×) and scanning electron microscope (2500×). When seeded onto uncoated glass surfaces, miR-200b- and miR-200c-overexpressing cells exhibited a rounded morphology as compared to the flat and extended morphology of miR-200a -overexpressing cells and empty vector controls. (**b**) Time-lapse microscope monitored the cell seeding during the first 24 hours. A delayed cell-substratum attachment was observed in miR-200b- and miR-200c-overexpressing cells. (**c**) Computer-aided cell migration path-tracking revealed a remarkable impairment of non-directional cell migration in miR-200b- and miR-200c-overexpressing cells. Lines representing the cell migratory paths of individual cells from their original positions (center).

The identification of RhoA and ROCK2 as the miR-200b subfamily targets prompted us to further investigate the roles miR-200b subfamily in HCC cytoskeletal reorganization. In this regard, immunofluorescence staining was performed to visualize the effects of miR-200 family on Rho/ROCK mediated stress fiber and focal adhesion formations. We found that the formations of stress fibers (stained by Phallodin) and focal adhesions (stained by anti-paxillin antibody) were profoundly impeded in miR-200b- and miR-200c-overexpressing cells as compared to the empty vector and miR-200a-overexpressing controls (Figure 4a and 4b). The loss of stress fiber and focal adhesion complex resulted in dramatic morphological change of the miR-200b and miR-200c overexpressing cells (Figure 4), and the morphological change could be more clearly visualized under the scanning electron microscope (SEM) (Figure 5a). The miR-200b and miR-200c overexpressing cells exhibited a rounded morphology, in contrast to a flat and extended morphology of the empty vector and miR-200a overexpressing cells (Figure 5a lower panel). Notably, the rounded cell morphology was more apparent when the cells were seeded on an uncoated glass coverslip as compared to the conventional “cell attachment enhanced” tissue culture plates. We therefore hypothesized that such morphological change reflected a loss of cell-substratum attachment ability and consequently hampered the cell motility of the miR-200b subfamily overexpressing cells. To address this question, we employed live cell imaging to monitor the cell-substratum attachment and non-directional random cell migratory abilities of BEL7402 cells. We found that the empty vector and miR-200a overexpressing cells were readily seeded onto the uncoated glass surface, exhibited an extended cell morphology and started to migrate within 12 hours, whereas miR-200b and miR-200c overexpressing cells failed to seed on the uncoated glass surface till the end of the experiment (Figure 5b and Supplementary Videos). Computer-aided cell migration tracking revealed that the cell migratory ability was modestly impaired in miR-200a overexpressing cells when compared to the empty control, but was drastically abolished in miR-200b and miR-200c overexpressing cells (Figure 5c). These findings collectively demonstrated the effects of the miR-200b subfamily in regulating Rho/ROCK-mediated cell cytoskeletal reorganization and provided a mechanistic explanation to the specific tumor suppressive functions of the miR-200b subfamily in HCC.

### MiR-200b suppressed HCC tumorigenicity and lung metastasis in mouse model

**Figure 6:**
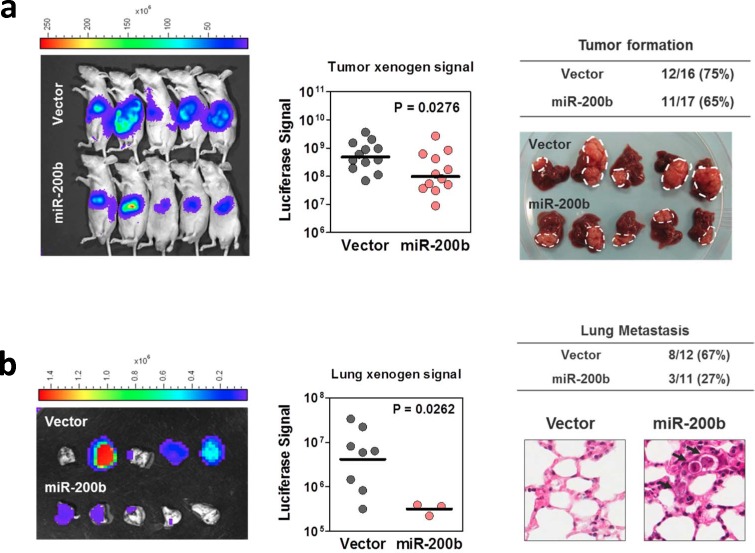
MiR-200b suppressed HCC tumorigenicity and lung metastasis in mouse model (**a**) Vector control and miR-200b stably overexpressing, luciferase-labelled MHCC97L cells were orthotopically implanted into the livers of the nude mice. After 4 weeks of inoculation, tumor formation was detected and quantified under *in vivo* bioluminescence imaging. Gross pathological examination was done on the tumor-bearing livers. Overexpression of miR-200b significantly suppressed the size of orthotopic tumors formed in nude mice. (**b**) The presence of lung metastasis was examined and quantitatively assessed in *ex vivo* bioluminescence imaging. Histopathological examination (H&E stain) was performed to confirm the presence of tumor foci formed in the lungs. Overexpression of miR-200b dramatically abolished the incidence and severity of lung metastasis in this *in vivo* orthotopic implantation model.

Since miR-200b subfamily was frequently down-regulated in human HCC samples and negatively regulated HCC cell proliferation and migration in *in vitro* experiments, we sought to investigate whether overexpression of miR-200b is sufficient to suppress *in vivo* tumorigenicity and extrahepatic metastasis in mouse model. In this experiment, miR-200b and empty vector control were stably infected into luciferase labeled MHCC97L cells, which had a low miR-200b expression and exhibited frequent lung metastasis when orthotopically implanted into the livers of nude mice [17]. After 4 weeks of implantation, the size of tumors formed by the miR-200b- overexpressing cells was significantly smaller that than of the vector controls (Figure 6a), although the rate of tumor formation was comparable between these two groups (75% [12/16] in vector control group versus 65% [11/17] in the miR-200b-overexpressing group). Importantly, lung metastasis, as evidenced by *ex-vivo* bioluminescent imaging and histopathological analysis, was drastically reduced in miR-200b-overexpressing group. Lung metastasis was detected in only 27% (3/11) of the tumor bearing mice in the miR-200b overexpressing group, whereas, in the control group, 67% (8/12) of the tumor bearing mice developed lung metastasis. Moreover, the number of metastasized cells, as quantified by the bioluminescence, was also significantly reduced upon overexpression of miR-200b (Figure 6b). The data obtained from this *in vivo* orthotopic implantation model echoed the findings of our *in vitro* studies and together provided strong evidence to support the tumor suppressive functions of miR-200b subfamily in human HCC.

## DISCUSSION

In this study, we demonstrated that all five members of miR-200 family (miR-200a, miR-200b, miR-200c, miR-141 and miR-429) were frequently down-regulated in human HCCs as compared to their corresponding non-tumorous livers. Consistent with our findings, down-regulation of miR-200 family has been previously observed in HCC miRNA profiling and miR-200 expression studies reported by others [18-20], suggesting that loss of miR-200 expression is a frequent event in liver carcinogenesis. MiR-200 family members are conventionally divided into two groups based on their genome locations clustered at 1p36 (miR-200a, miR-200b and miR-429) and 13p12 (miR-200c and miR-141), respectively. MiR-200s in each cluster are likely co-regulated by a common promoter and may express as one polycistronic primary miRNA transcript [9]. In fact, we observed that the expression levels of mature miR-200a and miR-200b, both from the 1p36 cluster, significant correlated with each other in both non-tumorous livers and primary HCCs (R^2^ = 0.691 and 0.603, respectively, *P* < 0.001, linear regression). The down-stream targets to be regulated by a given miRNA are very much determined by the miRNA seed-sequence (nucleotide 2-8). The down-stream target repertoire of a miRNA collectively defines its functions within a specific cellular content [21]. In this sense, the miR-200 family can be sub-divided into the functionally miR-200a subfamily (miR-200a and miR-141) and miR-200b subfamily (miR-200b, miR-200c and miR-429). *In silico* analyses have revealed that the down-stream targets of miR-200a and miR-200b subfamilies are largely non-overlapping with each other. Interestingly, we found that only the miR-200b subfamily members exerted tumor suppressive functions in HCC cells. Overexpression of miR-200b and miR-200c moderately suppressed HCC cell proliferation and colony formation, and drastically attenuated cell migration; all these effects were not seen in miR-200a overexpressing cells. These observations have raised two important issues. First, although the miR-200 family members are generally considered as functionally redundant paralogs, the miR-200a and miR-200b subfamilies in fact have distinct functions in regulating HCC cell growth and migration. Co-expression of two functionally distinct miRNA subfamilies in a primary miRNA transcript may suggest a new level of regulation or evolutionary selection. Whether functional cross-talks exist between miR-200 subfamilies in both normal cells and during pathological conditions is an interesting question worthy to be further explored. Second, both miR-200a and miR-200b subfamilies share the negative regulatory function on ZEB1/2 but only miR-200b subfamily suppressed HCC growth and migration, which implies that mechanisms in addition to the well-characterized miR-200s/ZEB1/2/E-cadherin axis may be involved to empower tumor suppressive functions specific to the miR-200b subfamily in HCC.

Attempting to address this question, *in silico* analyses were performed. In a pathway analysis, we found that down-stream targets of the miR-200b subfamily were specifically enriched in cytoskeleton proteins and participated in regulating small GTPase-mediated signal transduction. Among the candidate down-stream targets, we hypothesized that Rho-family small GTPase RhoA and its down-stream effector ROCK2 might be important for the miR-200b subfamily specific functions in HCC. RhoA and ROCK2 are the center of Rho/ROCK signaling pathway and are indispensable for actomyosin contraction, formation of stress fibers, and turnover of focal adhesions, thereby regulating cell movement [22, 23]. The activity of RhoA is determined by its GTP binding status and can cycle between GTP-bound active and GDP-bound inactive forms through its intrinsic GTP hydrolysis activity. Activated RhoA binds to and releases auto-inhibitory structure of its effector kinases, ROCK1 and ROCK2, and in turn, induces phosphorylation of multiple cytoskeletal proteins [22, 23]. The Rho/ROCK signaling pathway is frequently altered in human HCC by multiple mechanisms and consequently implicated in HCC metastasis. We previously reported that the RhoA up-stream regulators, DLC1 and DLC2 (deleted in liver cancer 1 and 2), were frequently down-regulated in human HCC by genetic deletion and epigenetic silencing [24, 25]. DLC1 and DLC2 are Rho-GTPase activating proteins (RhoGAP) which inactivate RhoA by promoting its GTP hydrolysis [24, 25]. Loss of DLC1 and DLC2 expression therefore leads to the hyperactivation of Rho/ROCK signaling pathway in human HCC [26]. In addition, we reported that ROCK2 but not ROCK1 was specifically up-regulated in human HCC, which highlights the importance of ROCK2 in Rho/ROCK-mediated HCC tumorigenesis and metastasis [27]. More recently, we have further demonstrated that the frequent up-regulation of ROCK2 was partially contributed by the loss of expression of its regulatory miRNA, miR-139 [17]. In the present study, using luciferase reporter assay, qRT-PCR, and Western blotting, we confirmed that the miR-200b sub-family interacted with RhoA and ROCK2 3′UTR and negatively regulated RhoA and ROCK2 expression at both mRNA and protein levels. Overexpression of the miR-200b subfamily significantly suppressed Rho/ROCK mediated cytoskeletal reorganization, as evidenced by the loss of stress fiber and focal adhesion formations, and induced a dramatic cell morphological change. This phenotype was similar to the morphological change we observed in RhoA and ROCK2 knockdown cells (Supplementary Figures 7 and 8), strongly suggesting that this miR-200b-mediated effect was attributed to the inactivation of Rho/ROCK mediated cytoskeletal reorganization. We further demonstrated by live cell imaging that inactivation of Rho/ROCK signaling, through miR-200b subfamily overexpression, attenuated cell-substratum adhesion and consequently hampered cell migration. Altogether, these findings have uncovered a novel function of miR-200b subfamily in regulating Rho/ROCK mediated cytoskeletal reorganization and cell motility.

Finally, we employed an orthotopic implantation model to further demonstrate the functions of miR-200b in suppressing HCC tumorigenicity and metastasis *in vivo*. This model allows the HCC cells to form a tumor in a native liver microenvironment and metastasize to distant organs. The presence of luciferase reporter also facilitates the monitoring and quantitative measurement of orthotopic tumor growth and extra-hepatic metastasis in the mice. As expected, overexpression of miR-200b resulted in a modest inhibition of HCC cell growth in hepatic microenvironment. Importantly, the formation of lung metastasis was drastically abolished upon miR-200b overexpression. These findings coherently echoed with our *in vitro* findings and together confirmed the tumor suppressive functions of miR-200b subfamily in HCC.

In summary, the work present here revealed that all the miR-200 family members were frequently down-regulated in human HCC. Only the miR-200b subfamily but not its closely related miR-200a subfamily possessed tumor suppressor functions in HCC. The identification of RhoA and ROCK2 as the miR-200b subfamily specific targets highlighted the novel function of the miR-200b subfamily in regulating cytoskeletal organization. Our findings have demonstrated the functional discrepancy of miR-200 family members and provided a mechanistic explanation toward the miR-200b specific tumor suppressive function in HCC.

## MATERIALS AND METHODS

### HCC patients and cell lines

Sixty-four patients who had primary HCC were involved in this study. Seventy-five percent of the patients had chronic HBV infection; the mean age was 54 ± 13 years. The HCC and their corresponding non-tumorous liver samples were obtained from surgical resection at Queen Mary Hospital, Hong Kong. Normal liver samples were collected from patients who had resection of liver metastasis from colorectal carcinoma. Samples were immediately snap-frozen in liquid nitrogen and stored at −80°C for later use. Written consent was obtained from patients regarding the use of the specimens for scientific research. The use of clinical specimens for this study was approved by Institutional Review Board of the University of Hong Kong and the Hospital Authority of Hong Kong.

Immortalized hepatocyte cell line, MIHA and human HCC cell lines, BEL7402 and SMMC-7721, were obtained from the Shanghai Institute of Cell Biology. Hep3B and HepG2 were purchased from American Type Culture Collection (ATCC). H2P and H2M cell lines were gifts from Dr XY Guan (the University of Hong Kong). Metastatic MHCC97L used for orthotopic implantation experiment was obtained from Dr. ZY Tang (Fudan University, Shanghai, China) and we labelled it with fire-fly luciferase reporter as previously described [17].

### MicroRNA profiling

MiRNA profiling was performed on 20 HCC patients with TaqMan Low-density array human microRNA set 2 (Applied Biosystems) and has been reported previously [4]. In berify, miRNA was extracted from formalin-fixed, paraffin-embedded (FFPE) HCC and non-tumorous liver samples using miRNeasy FFPE kit (Qiagen, Valencia, CA). Mature miRNAs were reverse transcripted and pre-amplified with Megaplex-Pre-Amp reaction. Real-time qunatitative PCR was then conduced with TaqMan Low density Array (TLDA) Human microRNA version 2, as described in the manufacturer's protocol (Applied Biosystems).

### Quantitative reverse transcription polymerase chain reaction (qRT-PCR)

Total RNA was extracted from clinical samples and cell lines using TRIZOL reagent (Life technologies). For protein coding genes and primary miRNAs, complementary DNA (cDNA) was synthesized from 1μg of total RNA using the GeneAmp RNA PCR kit (Life Technologies) with random hexamer primers. PCR reactions were performed in ABI 7900HT Fast Real-Time PCR systems (Applied Biosystems) with the Power SYBR® Green Master Mix (Life technologies). For mature miRNAs, cDNA was synthesized from 10ηg of total RNA using the TaqMan MicroRNA Reverse Transcription Kit with miRNA specific stem-loop primers (Applied Biosystems). PCR reactions were performed in triplicate in 7900HT Fast Real-Time PCR systems (Applied Biosystems) with specific TaqMan MicroRNA assays (Applied Biosystems). GAPDH and RNU44 were used as internal controls to normalize the relative expression levels of the protein coding genes and mature miRNAs, respectively.

### MiR-200 family miRNA precursors and LNA inhibitors

1× 10^5^ cells were seeded in 12-well plate a day before transfection. For miR-200 overexpression, the cells were transfected with 30 ηM of designated miRNA precursors or pre-miR negative control 1 (Applied Biosystems) using X-tremeGENE siRNA transfection reagent (Roche). For miR-200 inactivation, cells were transfected with 50 ηM designed miRCURY LNA™ miRNA Inhibitor or LNA control (Exiqon).

### Establishment of miR-200s stably expressing cells

*MiR-200a*, *miR-200b* and *miR-200c* genes were amplified from the genomic DNA of MIHA cells and sub-cloned into a lenti-viral based miRNA expression vector, pCDH-CMV-MSC-EF1-copGFP/Puro (System Biosciences) utilizing the EcoRI and NotI restriction sites. Lenti-viral particles were packaged in 293FT cells with pPACK Packaging Systems (System Biosciences) and used to infect BEL7402, SMMC-7721 and luciferase-labelled MHCC97L cells. Infected cells were then selected by 1μg puromycin for two weeks. The successful overexpression and post-translational gene regulatory activity of miR-200 family members in the stably infected cells were confirmed by qRT-PCR and luciferase reporter assays.

### Cell proliferation and colony formation assays

For cell proliferation assay, 1× 10^5^ cells were seeded in 12-well plate a day before cell counting. The cell number was counted daily with Particle Counter (Beckman Coulter, Fullerton, CA) for five days. For colony formation assay, 5,000 cells were seeded onto 6-well plates coated with soft agar. After incubation in a humidified incubator at 37°C for a month, the number of HCC cell colonies (> 100μm) formed was counted and pictured under microscope.

### Cell migration assay

Migration assay was performed with 8.0μm-pore-size Transwell inserts (Corning Inc., NY). 1× 10^5^ cells resuspended in serum-free medium were seeded onto the upper chamber, and culture medium containing 10% FBS was added to the lower chamber as chemoattractant. After incubation in a humidified incubator at 37°C for 24 hr, the migrated cells were fixed, stained, and counted under microscope.

### *In silico* miRNA target prediction

MiRNA target prediction and Gene ontology (GO) analysis were performed using EIMMO miRNA prediction server (http://www.mirz.unibas.ch/ElMMo3/) [16]. The candidature of RhoA and ROCK2 as miR-200b subfamily specific targets was further evaluated by TargetScan5 (http://www.targetscan.org) [28] and miRanda (http://www.microrna.org) [29] algorithms.

### Luciferase reporter assay

Wild-type and mutated miR-200b subfamily targeting sites on RhoA (132-153 nt.) and ROCK2 (1005-1026 nt.) 3′UTR were cloned into a Dual-luciferase miRNA target expression vector pmirGLO (Promega). In addition, miRNA specific sensors containing the complementary sequences to individual mature miR-200 family members were also cloned into the same vector and served as positive control for accessing the post-translational gene repression activity of ectopically expressed miR-200 family members. 5 × 10^4^ HCC cells were seeded into each well of a 24-well plate the day before transfection. 15 ηM of each miR-200 precursors was first individually transfected into HCC cells using X-tremeGene. Twenty-four hours later, 0.5 μg of pmirGLO containing RhoA or ROCK2 3′UTR sequence was then transfected into the cells using FuGENE 6 (Roche). The firefly and *Renilla* luciferase activities of transfected cells were determined 48 hours after transfection using Dual-luciferase Assay Kit (Promega) according to the manufacturer's protocol. *Renilla* luciferase activity was used as internal control for normalization.

### Protein extraction and western blotting

Cells were lysed with 1% NP40 NET buffer for total protein extraction. Proteins were resolved in SDS-PAGE and blotted onto nitrocellulose membrane. The membrane was incubated with primary antibody overnight at 4°C, followed by anti-mouse immunoglobulin G (GE Healthcare) for one hour at room temperature. The ECL detection system (GE Healthcare) was used for protein detection according to the manufacturer's protocol. Anti-E-cadherin and anti-ROCK2 antibodies were purchased from Santa Cruz Biotechnology, anti-RhoA antibody from Cytoskeleton and anti-β-actin antibody from Sigma-Aldrich.

### Immunofluorescence staining and scanning electron microscopy

1 × 10^4^ cells were seeded on glass coverslips in 12-well plates. After seeding, cells were serum starved for 24 hr. Cells were treated with 10μM lysophosphatidic acid for 30 minutes and then fixed with 4% paraformaldehyde in PBS and permeabilized with 0.2% Triton-X-100. F-actin was visualized by staining with TRICT conjugated phalloidin (Sigma Aldrich). Focal adhesions were stained with anti-paxillin antibody (Millipore). Nuclei were counter-stained with DAPI (Calbiochem). Immunofluorescence images were captured under Leica Q550CW fluorescence microscope (Leica) at 100× magnification. For scanning electron microscopy, cells were fixed with 2.5% glutaraldehyde and 1% osmium tetroxide. Fixed cells were then subjected to stepwise ethanol dehydration and critical point drying. Images were scanned and captured under 2,500× magnification using a Hitachi S-4800 FEG Scanning Electron Microscope (Hitachi).

### Time-lapse microscopy

Cells were plated in 12-well glass-bottomed imaging plates (MatTek) and cultured in CO_2_-independent medium (Invitrogen). Cell images were acquired using the Nikon TE2000-PFS inverted microscope (Nikon) enclosed in a humidified chamber maintained at 37°C. Cells were imaged every 10 minutes immediately after cell seeding with a 20× objective on five randomly selected fields per sample. Videos were generated with MetaMorph software (Molecular devices). For cell tracking analysis, the migration paths of 100 cells per sample were traced between 18 to 72 hours after cell seeding. Data were analyzed by Image-Pro Plus 6.0 (MediaCybernetics).

### *In vivo* orthotopic implantation model

MiR-200b stably expressing cells were established from luciferase labelled MHCC97L cells [17]. 2 × 10^6^ cells were resuspended in 25 μL DMEM-HG/Matrigel (BD Biosciences) at 1:1 ratio and orthotopically injected into the left liver lobe of 6-8 weeks old male BALB/cAnN-nude mice. Mice were sacrificed at week 4 and *in vivo* tumor growth and lung metastasis were accessed by IVIS 100 Imaging System (Xenogen). All animal experiments were performed according to the Control of Animals Experiments Ordinance (Hong Kong) and the Institute's guidelines on animal experimentation.

### Statistical analysis

Statistical analyses were performed using PRISM5 software (GraphPad). P values less than 0.05 were considered statistically significant.

## SUPPLEMENTARY MATERIAL, FIGURES, TABLE AND VIDEOS











## Abbreviations

HCC: Hepatocellular carcinoma
miRNA: MicroRNA
EMT: Epithelial-mesenchymal transition
ZEB1/2: Zinc Finger E-Box Binding Homeobox 1/2
MET: Mesenchymal-epithelial transition
qRT-PCR: Quantitative reverse transcription polymerase chain reaction
GO: Gene ontology
RhoA: Ras homolog family member A
ROCK2: Rho-associated, coiled-coil containing protein kinase 2
LNA: Locked nucleic acid
UTR: Untranslated region
SEM: Scanning electron microscope.
