# Supplementary material for: MiR-200b/200c/429 subfamily negatively regulates Rho/ROCK signaling pathway to suppress hepatocellular carcinoma metastasis
Source: Oncotarget. 2015 Mar 30;6(15):13658–70. doi: 10.18632/oncotarget.3700 (PMC4537040; doi:10.18632/oncotarget.3700)
Supplement: Supplementary file 1 [file oncotarget-06-13658-s001.pdf]

MiR-200b/200c/429 subfamily negatively regulates Rho/ROCK signaling pathway to suppress hepatocellular carcinoma metastasis

Supplementary Material

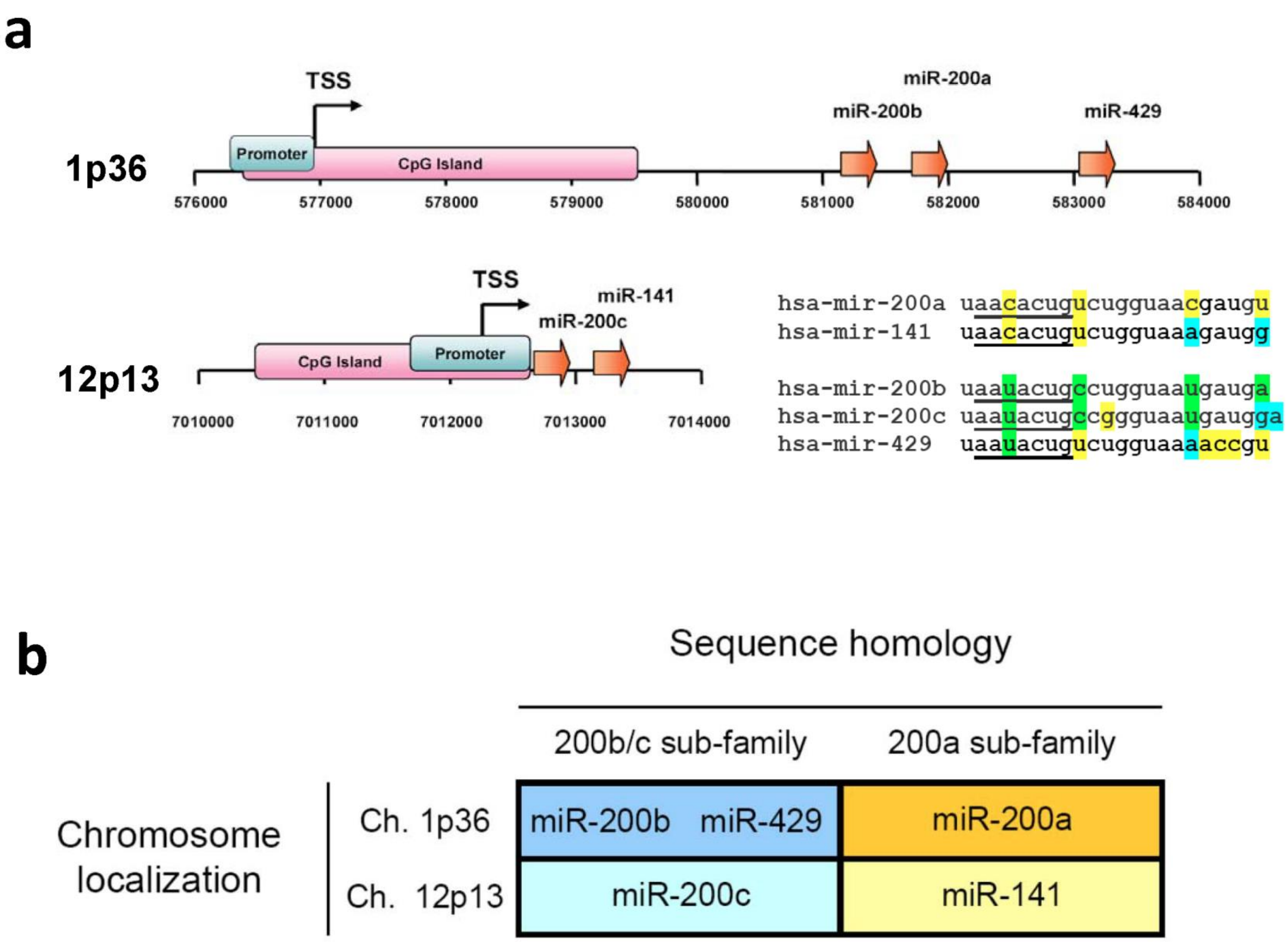

Supplementary Figure 1: Chromosomal location and sequence homology of the miR-200 family members. **(a)** Chromosomal position was based on Genebank record NT_004350 and NT_009759. MiRNA seed sequences were underlined and sequence variants among the miR-200 family members were highlighted with colored boxes. **(b)** The miR-200 family can be subdivided in two groups, either by their chromosome location or their seed sequence homology. The miR-200 family members at the same cluster are co-regulated by their upstream promoter, whereas their functions are determined by their seed-sequence homology.

H2P cell line: Primary HCC from a 42-years old male patient
H2M cell line: metastatic thrombosis in the portal vein from the same patient

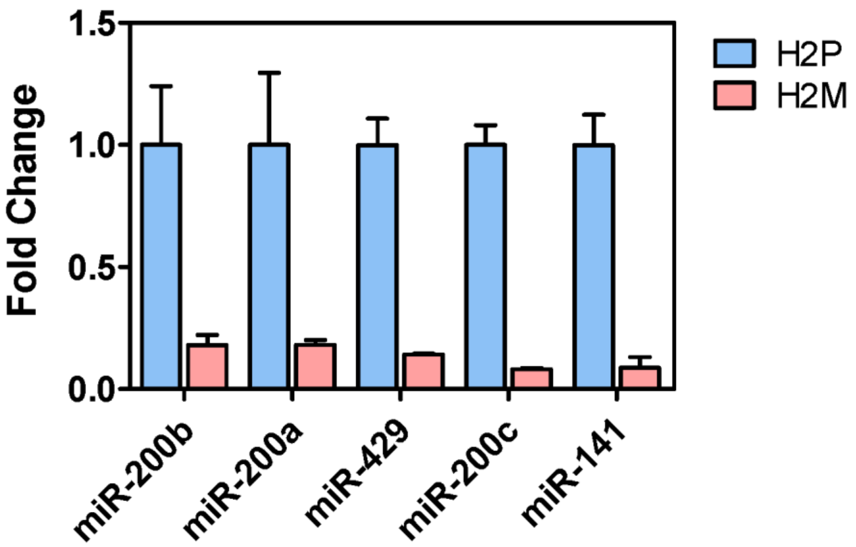

Supplementary Figure 2: Expression of the miR-200 family in a pair of HCC cell lines derived from the primary tumor (H2P) and portal vein metastasis (H2M) of the same patient. Expression levels of primary miR-200 transcripts were determined by qRT-PCR and normalized with GAPDH.

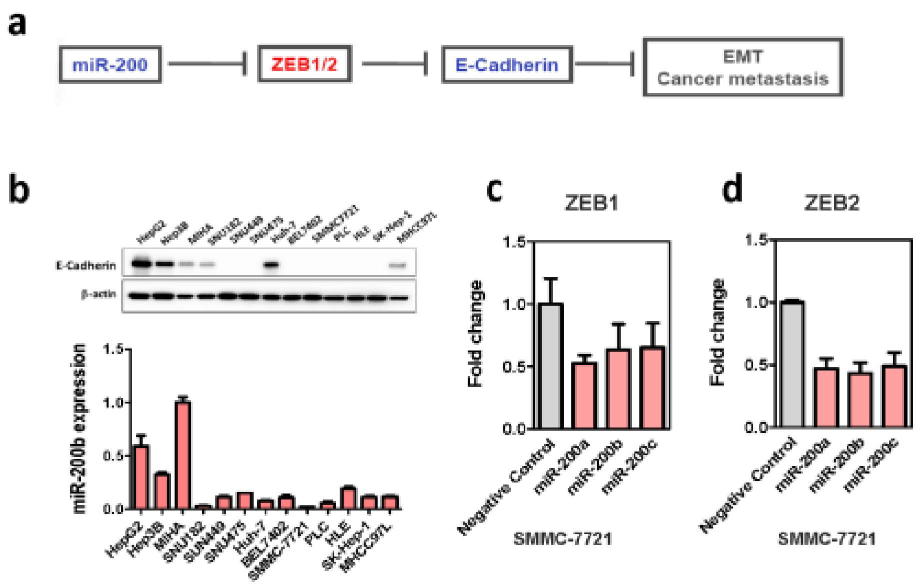

Supplementary Figure 3: The miR-200 family suppressed ZEB1 and ZEB2 expression in human HCC cells. (a) Schematic diagram illustrating the well characterized miR-200/ZEB1/2/E-cadherin axis in EMT and cancer metastasis. (b) The expression levels of E-cadherin and miR-200b in HCC cell lines correlated with each other. (c) Transient overexpression of miR-200a, miR-200b and miR-200c precursors all significantly suppressed the expression of ZEB1 and ZEB2 in HCC cell lines.

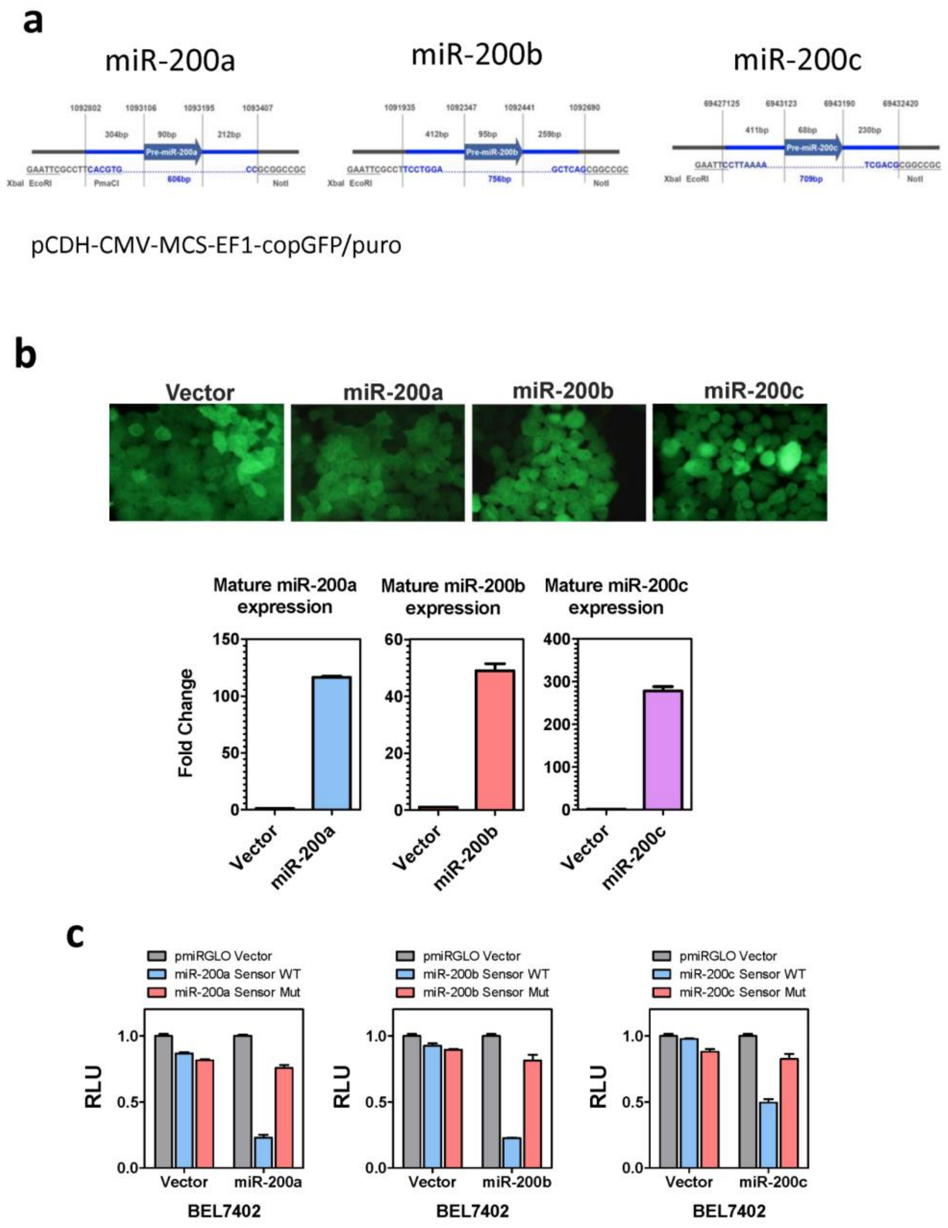

Supplementary Figure 4: Establishment of miR-200 stably overexpressing cells. **(a)** MiR-200a, miR-200b and miR-200c were amplified from MIHA genomic DNA and subcloned into a lenti-viral expression vector, pCDH-CMV-MSC-EF1-copGFP/puro (Systems Biosciences). **(b)** Successful stable overexpression of miR-200s was confirmed with GFP marker and qRT-PCR. **(c)** The transcriptional repressive activities of ectopically expressed miR-200s were confirmed by wild-type and seed-sequence mutated miRNA sensors (mature miRNA complementary sequences).

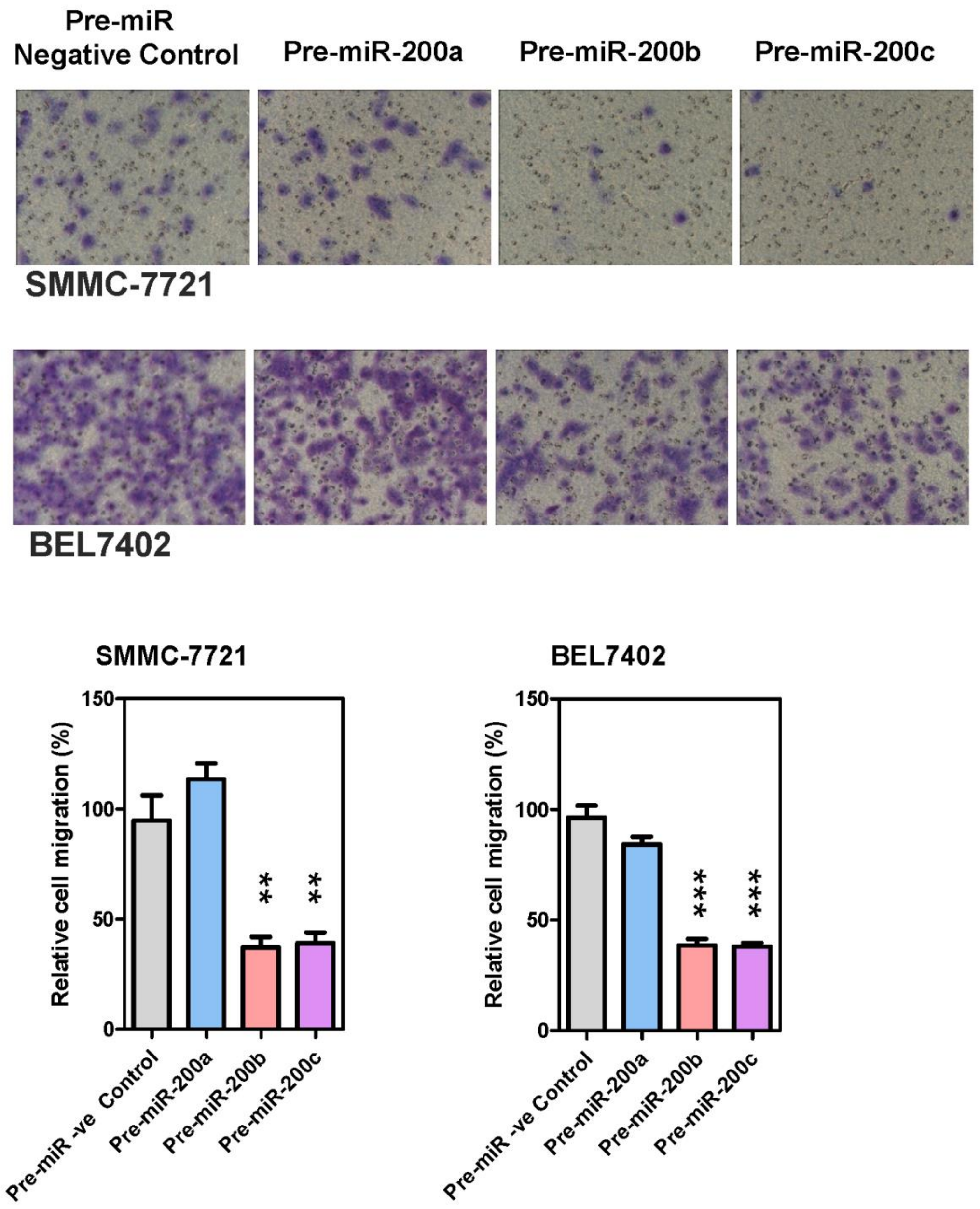

Supplementary Figure 5: Transient overexpression of miR-200b and miR-200c precursors suppressed HCC cell migration. HCC cells were transiently transfected with miR-200 precursors and their effect on cell migration was examined by Transwell cell migration assay. Consistent with the miR-200 stable overexpression model, expression of miR-200b and miR-200c (but not miR-200a) precursors significantly suppressed cell migration in both SMMC-7721 and BEL7402 cell lines. ** $P < 0.01$, *** $P < 0.001$, t-test.

	RhoA	
	miR-200a/141	miR-200b/200c/429
EIMMO	X	○
TargetScan	X	○
miRanda	X	○

	ROCK2	
	miR-200a/141	miR-200b/200c/429
EIMMO	X	○
TargetScan	X	○
miRanda	X	○

Supplementary Figure 6: MiRNA target prediction algorithms consistently predicted RhoA and ROCK2 as the specific down-stream targets of the miR-200b subfamily. ○: Predicted targets, ×: Non-targets.

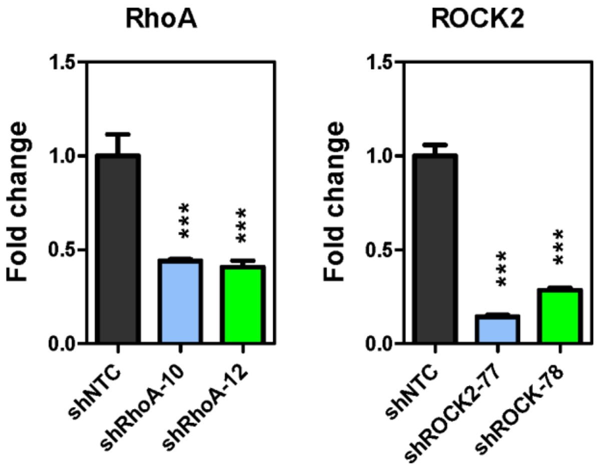

Supplementary Figure 7: Knockdown of RhoA and ROCK2 in BEL7402 cells by two independent shRNA sequences as verified by qRT-PCR.

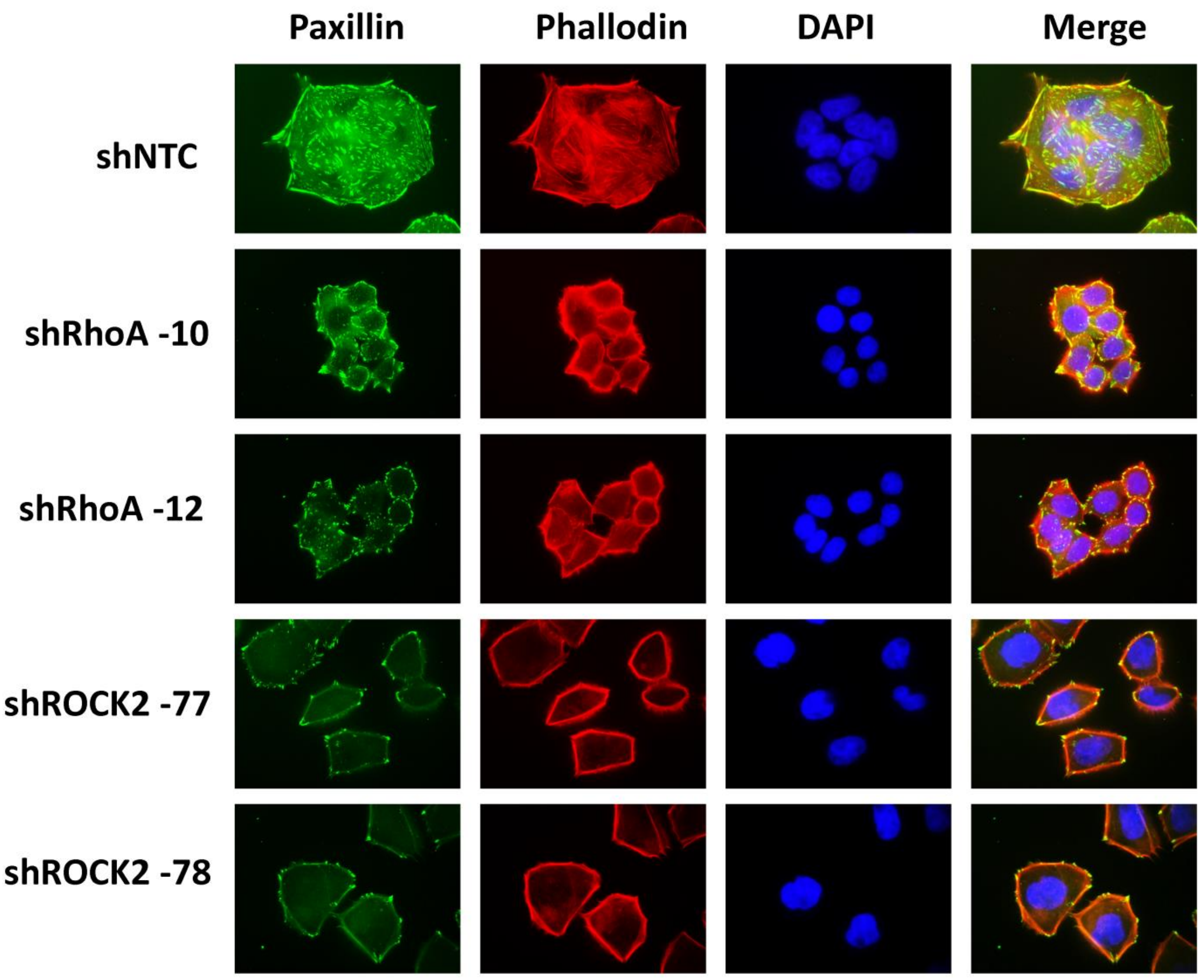

Supplementary Figure 8: Knockdown of RhoA (shRhoA-10 and -12) and ROCK2 (shROCK2-77 and -78) phenocopied miR-200b sub-family overexpression in suppressing stress-fiber and focal adhesion formation.

Supplementary Table 1: Gene ontology (GO) analysis performed by EIMMO miRNA prediction server.

Gene ontology (GO) analysis performed by EIMMO microRNA prediction server

	**miR-200a/141**	
	GO Term	Bonferonni corrected p-Value
Cellular component	nucleus	3.01E-12
	protein phosphatase type 2A complex	1.21E-03
	microtubule associated complex	2.11E-02
Biological process	protein amino acid phosphorylation	4.57E-09
	regulation of transcription from RNA polymerase II promoter	4.84E-05
	regulation of transcription, DNA-dependent	2.46E-03
	morphogenesis	1.97E-02

	**mIR-200b/200c/429**	
	GO Term	Bonferonni corrected p-Value
Cellular component	nucleus	2.47E-11
	cytoskeleton	1.15E-05
	voltage-gated sodium channel complex	1.85E-03
	ubiquitin ligase complex	9.40E-03
	nuclear inner membrane	1.87E-02
	euchromatin	1.87E-02
	nuclear matrix	2.61E-02
	Golgi apparatus	3.61E-02
Biological process	regulation of transcription, DNA-dependent	7.23E-08
	protein amino acid phosphorylation	8.66E-06
	small GTPase mediated signal transduction	4.30E-03
	intracellular signaling cascade	1.40E-02
	regulation of transcription from RNA polymerase II promoter	1.55E-02

Common
miR-200a/141 specific
miR-200b/200c/429 specific

Supplementary Video 1: BEL7402 vector stable overexpressing cells

Supplementary Video 2: BEL7402 miR-200a stable overexpressing cells

Supplementary Video 3: BEL7402 miR-200b stable overexpressing cells

Supplementary Video 4: BEL7402 miR-200c stable overexpressing cells
